# Root traits predict decomposition across a landscape-scale grazing experiment

**DOI:** 10.1111/nph.12845

**Published:** 2014-05-20

**Authors:** Stuart W Smith, Sarah J Woodin, Robin J Pakeman, David Johnson, René van der Wal

**Affiliations:** 1 IBES, University of AberdeenSt Machar Drive, Aberdeen, AB24 3UU, UK; 2 The James Hutton InstituteCraigiebuckler, Aberdeen, AB15 8QH, UK; 3 ACES, University of AberdeenSt Machar Drive, Aberdeen, AB24 3UU, UK

**Keywords:** carbon (C), grassland, grazing, nitrogen (N), plant traits, root decomposition, soil moisture, soil temperature

## Abstract

- Root litter is the dominant soil carbon and nutrient input in many ecosystems, yet few studies have considered how root decomposition is regulated at the landscape scale and how this is mediated by land-use management practices. Large herbivores can potentially influence below-ground decomposition through changes in soil microclimate (temperature and moisture) and changes in plant species composition (root traits).
- To investigate such herbivore-induced changes, we quantified annual root decomposition of upland grassland species *in situ* across a landscape-scale livestock grazing experiment, in a common-garden experiment and in laboratory microcosms evaluating the influence of key root traits on decomposition.
- Livestock grazing increased soil temperatures, but this did not affect root decomposition. Grazing had no effect on soil moisture, but wetter soils retarded root decomposition. Species-specific decomposition rates were similar across all grazing treatments, and species differences were maintained in the common-garden experiment, suggesting an overriding importance of litter type. Supporting this, in microcosms, roots with lower specific root area (m^2^ g^−1^) or those with higher phosphorus concentrations decomposed faster.
- Our results suggest that large herbivores alter below-ground carbon and nitrogen dynamics more through their effects on plant species composition and associated root traits than through effects on the soil microclimate.

## Introduction

Regulation of plant litter decomposition determines carbon (C) and nitrogen (N) cycling in soils. Litter decomposition rates are influenced by a range of biological and environmental factors, including litter quality – the availability of nutrients and their ratios within the litter – and, importantly, edaphic factors such as soil moisture and temperature. Our understanding of litter decomposition is almost exclusively based on studies of above-ground plant material (Zhang *et al.*, 2008; Prescott, 2010; Freschet *et al.*, 2013), but the dominant plant inputs into soil in many ecosystems are below ground (Gill & Jackson, 2000). For example, in temperate grasslands, C inputs from roots can be up to three times greater than above-ground inputs (Robinson, 2007; Freschet *et al.*, 2013). By decomposing in the soil rather than on the soil surface, roots remain in a relatively stable decomposition environment compared with above-ground plant litter exposed to fluctuations in temperature and moisture (Silver & Miya, 2001; McLaren & Turkington, 2010). The assumption that root and leaf decomposition rates are comparable and equally responsive to the processes controlling decomposition, such as climatic conditions, may lead to erroneous predictions of C cycling (see Freschet *et al.*, 2013). This commonly held assumption underlying models currently used to predict soil C stocks (Smith *et al.*, 1997; Jones *et al.*, 2005; Davidson & Janssens, 2006) is challenged by the fact that the soil environment buffers climatic effects on root decomposition.

At larger spatial scales, root decomposition may be influenced not only by climatic factors but also by land management practices such as herbivore stocking rates. Large herbivores affect organic matter decomposition and its regulatory processes (Bardgett *et al.*, 1998; Piñeiro *et al.*, 2010); thus grazing intensity can potentially be used as a management tool to influence C storage in grassland and rangeland systems (Jones & Donnelly, 2004; Piñeiro *et al.*, 2010; Tanentzap & Coomes, 2012). Herbivores consume the plant canopy, allowing greater radiative energy to reach the soil and simultaneously reducing the transpiration surface area and therefore water losses (Moretto *et al.*, 2001; Piñeiro *et al.*, 2010; Klumpp *et al.*, 2011). This creates a warmer and wetter soil microclimate, which ought to favour root decomposition. However, empirical evidence of grazing-induced changes in soil temperature and moisture influencing root decomposition is ambiguous. For example, in semi-arid grasslands, increased grazing pressure has been shown either to enhance root decomposition (Shariff *et al.*, 1994) or to have no significant effect despite changing soil temperature or moisture (Moretto *et al.*, 2001). In montane grassland soil, temperature, moisture and decomposition were unaffected by grazing, but buried cotton-strip decomposition rates could be partially explained by landscape-scale variation in soil microclimate (Risch *et al.*, 2007). It remains unclear whether, in a grazed landscape, the influence of grazing on soil microclimate is less important than landscape heterogeneity of edaphic factors.

Both grazing pressure and plant community distribution are heterogeneous in the landscape and both may drive below-ground processes. Herbivores may indirectly affect root decomposition through modifying plant communities and thereby the decomposition environment. Selective grazing of palatable species affects plant canopy structure, community composition and biomass distribution, all of which influence the decomposer community (Holland *et al.*, 1992; Bardgett *et al.*, 1998; Wardle *et al.*, 2004; Klumpp *et al.*, 2009). The effects of individual plant species on microbial degradation of root litter vary depending on the release of labile C compounds from live roots (Van der Krift *et al.*, 2001, 2002), the supply of oxygen in anaerobic soil through aerenchyma (Weiss *et al.*, 2005; Neubauer *et al.*, 2007) and the desiccation of the soil as a result of plant water use (Jenkinson, 1977). The dominant plant species of a sward can support a microbial community that decomposes its own litter faster than litter originating from different species from another area (‘home-field advantage’) (Ayres *et al.*, 2009; Freschet *et al.*, 2012b). Plant community or individual plant species' effects on root decomposition may outweigh the effects of grazing, or these two factors may interact. However, this remains a moot point, as few studies have attempted to untangle the relative importance of plant species on root decomposition in grazed systems.

Rates of root decomposition for individual plant species depend on the quality of litter entering the soil. Variation in root quality is generally presumed to reflect patterns observed for leaf litter. At one end of the spectrum are palatable species with high N, calcium (Ca), potassium (K) and phosphorus (P) content, high specific leaf area and low lignin and recalcitrant C compound contents. At the opposite end of the spectrum are nutrient-conservative species with unpalatable, tough leaves with low nutrient contents and abundant recalcitrant C compounds (Grime *et al.*, 1997; Cornwell *et al.*, 2008; Orwin *et al.*, 2010; Freschet *et al.*, 2013). Studies investigating root decomposition have found that species differences are similarly predicted by some of these traits, for example, hemicellulose and cellulose content, P and root specific length (Personeni & Loiseau, 2004; Vivanco & Austin, 2006; Birouste *et al.*, 2012). By contrast, some traits that predict decomposition of above-ground material, notably root N and Ca contents, appear to be inconsistent predictors of root decomposition (Silver & Miya, 2001; Hobbie *et al.*, 2010; Birouste *et al.*, 2012; Freschet *et al.*, 2012a). Another potentially important factor regulating root decomposition is the extent of colonization of roots by symbiotic mycorrhizal fungi. Langley *et al.* (2006) found that root decomposition rate was accelerated by the extent of colonization of decaying roots, similar to findings in grassland microcosm systems showing that arbuscular mycorrhizal fungi accelerate leaf litter decomposition (Hodge *et al.*, 2001). The majority of root trait analyses are from pot, microcosm or common-garden experiments under similar soil microclimate conditions. Therefore, to ascertain which traits are consistent predictors of root decomposition, they need to be studied in the field under a range of soil microclimates prevailing under plant communities.

Here we address the significant knowledge gap concerning the controls of root decomposition by investigating the relative influence of species traits, livestock grazing and landscape heterogeneity. We quantified root litter decomposition of four dominant upland graminoid species (*Agrostis capillaris*, *Juncus effusus*, *Molinia caerulea* and *Nardus stricta*) *in situ* across a landscape-scale grazing manipulation experiment established for *c*. 8 yr. In addition, key root traits of these four and a further seven upland grassland species were measured and their influence on root decomposition was evaluated in laboratory microcosms. This allowed testing of the hypotheses that livestock grazing influences decomposition of root litter indirectly via its effects on soil microclimate; that species root traits have greater influence on root decomposition than do soil microclimate and dominant vegetation type; and that root chemical and morphological traits can be used to predict root decomposition.

## Materials and Methods

### Field site and experimental design

The field decomposition study was undertaken at Glen Finglas in central Scotland (56°16′N 4°24′W). This upland area (200–500 m above sea level (a.s.l.)) has mean annual rainfall of 1344 mm and mean January and July temperatures of 2.6 and 14.3°C, respectively (1982–2000 average from Loch Venachar at 5 km distance; UK Meteorological Office, 2012). Soils are organic and include blanket peats, peaty gleys and humus iron podzols, with 60% of the area having soil (to a depth of 15 cm) comprising > 40% C (Soil Survey of Scotland, 1984; SIFSS, 2013). The vegetation is a fine-grained mosaic of the following communities (British National Vegetation Classification codes in brackets; Rodwell, 1991, 1992): *Juncus effusus/acutiflorus*–*Galium palustre* rush-pasture (M23) and *Molinia caerulea*–*Potentilla erecta* mire (M25), both with a tall sward; and *Festuca ovina*–*Agrostis capillaris*–*Galium saxatile* grassland (U4) and *Nardus stricta*–*G. saxatile* grassland (U5), with shorter swards. The area is grazed by black-faced sheep and Luing cattle, typical of many upland areas of Scotland. Grazing is selective and thus grazing pressure is heterogeneous within the landscape. Plant height in *A. capillaris* and *N. stricta* communities is significantly reduced by grazers, whilst in *J. effusus* and *M. caerulea* dominated swards grazing reduces the abundance of tussocks without much effect on canopy height (Dennis *et al.*, 2004; Smith *et al.*, 2014).

In 2003, a landscape-scale grazing experiment was established across three sites, *c*. 4.1 km apart, within Glen Finglas, each containing two large replicate experimental blocks. Each block comprised four 3.3 ha fenced plots which were randomly assigned one of the following grazing treatments: ‘commercial’ stocking, nine sheep per plot, giving a typical commercial stocking rate for nutrient-poor rough upland grassland of 2.7 ewes ha^−1^; ‘low’ stocking, three sheep per plot or 0.9 ewes ha^−1^, one-third of the commercial rate; ‘mixed’ stocking, two sheep and two cattle per plot, giving the same off-take as ‘low’ sheep grazing; and no livestock. Sheep remained in the plots throughout the year, only being removed for normal farm operations and during periods of severe weather; cattle were present in the mixed treatment for 4 wk in late summer only. Before initiation of the study, Glen Finglas was grazed by black-faced sheep at a low intensity (0.7 ewes ha^−1^), similar to the ‘low’ sheep grazing treatment.

### Root litterbags for field experiments

Litterbags were used to estimate annual root decomposition for four dominant upland graminoid species: *A. capillaris* (L.), *J. effusus* (L.), *M. caerulea* (L.) Moench and *N. stricta* (L.). Roots were collected from soil-vegetation monoliths (20 cm soil depth) in June/July 2010 from the low-intensity sheep grazing treatment only, thereby eliminating any confounding effects of grazing intensity on root quality (Shariff *et al.*, 1994). A mixture of both live and dead roots was collected; studies have shown no significant differences in root quality in live compared with ‘killed off’ roots as a result of a lack of N and P resorption during root senescence (Aerts, 1990; Aerts *et al.*, 1992). Chemical and morphological root traits for each species were measured before roots were prepared for litterbags (see later). Roots were pooled by species, air-dried for 5 d at 21°C, coarsely chopped and mixed. Nylon-mesh litterbags (9.5 cm × 8.5 cm, mesh size 50 μm to prevent in-growth of living roots) were prepared for both the grazing and common-garden experiments, each containing a standard 0.2 ± 0.001 g of litter.

### Decomposition of root litter in grazing experiment

The effect of increasing livestock densities, mediated through soil microclimate, on root decomposition of the four species was investigated by burying 288 litterbags across the grazing experiment (four species × four grazing treatments × six blocks × three replicates per plot). Roots were buried under their respective plant species at locations selected at random from long-term vegetation survey points within plots (Dennis *et al.*, 2004). These were a minimum of 13 m apart to reduce spatial covariation in soil physicochemical properties (Marriott *et al.*, 1997). Litterbags were buried at a 45° angle to a depth of 5 cm below the soil surface, where the majority of root decomposition naturally occurs (Fitter *et al.*, 1998; Rasse *et al.*, 2005). Litterbags remained in the soil for 1 yr and were collected in August/September 2011.

Three spot measurements of soil temperature (Jenway microprocessor, Model 3100, Cambridge, UK) and moisture (Theta probe ML2, Delta-T, UK) were made adjacent to each litterbag at a depth of 5 cm in September 2010, April/May 2011 and August/September 2011. A soil moisture value of 0.0 m^3^ m^−3^ signifies completely dry soil and 1.0 m^3^ m^−3^ signifies water-saturated soil (Anon, 1999). The effect of upland topography on the soil microclimate was accounted for using a Topographic Exposure score (TOPEX) at a resolution of 0.1 km× 0.1 km generated from a digital elevation model (OS 2003) in ArcGIS 9.3.

### Decomposition of root litter in common-garden experiment

We used a ‘common-garden’ approach to determine the relative influence of root traits compared with soil microclimate and dominant vegetation type on root decomposition. For each root species, five litterbags were buried under a *M. caerulea* sward (the dominant vegetation type within the grazing experiment; Smith *et al.*, 2014) in one randomly selected area within an ungrazed plot (56°27′N 4°38′W; 3 m × 3 m area). Similar decomposition rates for all four species would provide evidence of soil microclimate/sward type being the key controlling factors of root decomposition, whereas species-specific root decomposition (at similar rates to those in the main grazing experiment) would point to differences arising from litter type and underlying root traits.

### Root trait microcosm experimental design

Studying just four species does not allow for the identification of root traits that could explain species differences in decomposition. We therefore undertook a more detailed study of 11 upland species, including the four used in the field experiments. Species selected as representative of *A. capillaris-*dominated communities were germinated from seed (Emorsgate, UK; Les Semences du Puy, France), grown for 6 months (July 2010–January 2011) outdoors at the University of Aberdeen, UK (57°17′N 2°10′W) and included: grasses *A. capillaris*, *Anthoxanthum odoratum* (L.), *F. ovina* (L.), *Holcus lanatus* (L.), *M. caerulea* and *N. stricta*; sedge *Carex nigra* (L.) Reichard; rush *J. effusus*; and forbs *Cerastium fontanum* (Baumg), *Ranunculus acris* (L.) and *Rumex acetosa* (L.). Plants were grown in monoculture in 17 cm× 17 cm× 11.5-cm-deep pots filled with a 1 : 1 : 1 mixture of peat (Sinclair, professional, UK) : sand : terra-green absorbent granules (Oil-Dri UK Ltd, Wisbech, UK), with 5 g (wet weight) of roots collected from *A. capillaris-*dominated communities to encourage mycorrhizal colonization; no nutrients were added. Live roots were harvested, washed clean, air-dried, pooled within species, coarsely chopped and mixed. For each species, eight nylon mesh litterbags (5 × 5 cm, mesh size 100 μm) were prepared containing a standard 0.5 ± 0.005 g of roots. Litterbags were smaller than those used in the field to fit inside Kilner jars. This caused some litterbags to bulge and the central width of litterbags was measured using a hand-held calliper (± 0.5 mm) and included in the statistical analysis as bulge size g^–1^ litter mass (cm g^−1^). Litterbags were stored in a desiccator at room temperature before incubation.

Root litter was incubated in 0.5 dm^3^ glass Kilner jars for 6 months (May 2011–October 2011). Each jar contained 200 g of fresh, coarsely sieved iron-podzolic soil collected from Glen Finglas (92.1 mg g^−1^ C, 5.26 mg g^−1^ N, 0.45 mg g^−1^ P, pH 4.2; 31.5% sand, 61.5% silt, 7.0% clay) and a single litterbag per microcosm buried 2 cm below the soil surface. Microcosms were maintained at 14.5°C (the highest recorded soil temperature for *A. capillaris-*dominated communities during 2010–2011), in the dark, inside a controlled-environment plant growth chamber (ConViron®, Winnipeg, MB, Canada). Soil moisture content was maintained at 60% water holding capacity by weighing the microcosms and adding distilled H_2_O every 2 wk to compensate for water losses. Microcosms were loosely sealed using the jar lid to reduce soil water loss in the growth chamber and allow gas exchange.

### Root trait analysis

A suite of chemical and morphological traits commonly used to predict plant decomposition rates were analysed on roots before both field and microcosm decomposition experiments. Morphological traits were determined first on 10 replicates of fresh root material before pooling the root stock. Roots saturated to water holding capacity (see Cornelissen *et al.*, 2003) were weighed wet and scanned to determine root surface and length using an Epson flatbed scanner (Expression 10000XL 1.8 V3.4 3.04) to create a 400 dpi image that was analysed using WinRhizo V2009a 32 bit (Regent Instruments Inc., Sainte-Foy, QC, Canada) (Birouste *et al.*, 2012). Scanned roots were then oven-dried for 48 h at 70°C and reweighed to determine specific root area (SRA; root surface/oven-dried mass; m^2^ g^−1^), specific root length (SRL; total root length/oven-dried mass; m g^−1^) and root dry matter content (oven-dried mass/water-saturated mass; g g^−1^). The percentage of root length colonization by mycorrhizal fungi was assessed on fresh roots using the line-intersect method after aniline blue staining (McGonigle *et al.*, 1990).

All tissue chemical analyses were conducted on three to six replicates of oven-dried (48 h at 70°C) and steel ball-milled (Smith *et al.*, 2013) root material using standard protocols. C and N concentrations were determined by elemental analysis (NA 1500 Series 2; Carlo-Erba, Stanford, CA, USA). Ca, P and K concentrations were determined by sulphuric acid/hydrogen peroxide digestion, followed by ammonium molybdate/ascorbic acid colorimetric determination using flow injection analysis (FIAstar spectrophotometer 5023; Tecator, Höganas, Sweden) for P, and flame atomic absorbance spectrometry (Atomic Absorption Spectrophotometer Analyst 100; Perkin Elmer, Waltham, MA, USA) for Ca and K. Silica concentrations were assessed using an alkaline sodium hydroxide/hydrogen peroxide digest followed by determination of concentrations using flow injection analysis (Carneiro *et al.*, 2007). Root lignin and lignin-like substances were assessed using a sulphuric acid digestion method with the remaining oven-dried, acid-insoluble residue operationally defined as the root lignin and lignin-like fraction (Woodin *et al.*, 2009). Lignin : N and C : N ratios were calculated.

### Multiple measures of root decomposition in field and microcosm experiments

To encapsulate the multiple processes occurring during decomposition, at the end of the decomposition period we measured heterotrophic respiration, enzyme activity and loss of mass, C and N from roots. Heterotrophic respiration was measured *ex situ* under controlled abiotic conditions using an infrared gas analyser (IRGA; LI-8100, Li-Cor Biosciences Inc., Lincoln, NE, USA). Roots were extracted from litterbags, sealed in a polythene bag with a moist paper towel and incubated at 11.5°C (field site mean) overnight to saturate roots to maximum water holding capacity (Cornelissen *et al.*, 2003). Roots were weighed wet and placed in a custom-made 50 ml universal tube closed chamber connected to the IRGA. CO_2_ accumulation was recorded over 90 s; root CO_2_-C efflux rates were calculated from the linear increase in CO_2_ concentration within the tube and expressed as μmol CO_2_-C g^−1^ root C min^−1^. Extracellular phenol peroxidase activity, which is involved in the breakdown of phenolic compounds in roots, was determined via a colorimetric assay using L-3, 4-dihydroxyphenylalanine as a substrate that produces dihydroindole-quinone-carboxylate (diqc) as a product; enzyme activity was expressed as μmol diqc g^−1^ min^−1^ (Papanikolaou *et al.*, 2010). For roots from the field, phenol peroxidase activity was extremely variable and was not included in the analysis. Mass, C and N contents of roots at the end of the decomposition period were determined by weighing and elemental analysis (as described earlier). Root decomposition was expressed as loss of DW mass, C and N from roots, divided by initial values (g g^−1^).

### Statistical analysis

#### Field root decomposition experiment analysis

Roots from 268 litterbags recovered after 1 yr of decomposition across all grazing treatments (out of 288) were used in statistical analysis conducted in R using the lme4 package (version 2.10.1, R Development Core Team, 2009; Bates & Maechler, 2010). The effects of grazing treatments on soil microclimate and root decomposition were explored using linear mixed-effect models with residual maximum likelihood estimations (REML). The random structure, reflecting the experimental design, was defined as plot nested within block nested within site. Soil microclimatic variables were averaged over time, as this explained more variation in decomposition than individual measurement dates. Six models were used: two to explore factors influencing soil temperature and soil moisture separately and four exploring different root decomposition measures (loss of mass, C and N and CO_2_-C efflux from roots). One-third of the roots recovered from the field did not produce a detectable CO_2_-C efflux, significantly zero-inflating the dataset. Undetectable fluxes were not a function of species identity and grazing treatment (*χ*^2 ^= 6.6, df = 3, *P *> 0.05). Therefore, only roots that produced a CO_2_-C efflux were analysed using a linear mixed model (*n* = 179). Soil microclimate and decomposition measures were analysed for the effect of the following in sequential order: grazing treatment, plant species, soil temperature, soil moisture, topographical exposures (TOPEX score) and all interactions with species and soil temperature and moisture.

Final models were simplified following Akaike's information criterion (AIC), removing terms from the full model to improve the model likelihood and lower AIC value. Fixed variables were retained if significant in likelihood ratio deletion tests (LRTs) (Pinheiro & Bates, 2000). For the final model, the significance of each term was assessed by removing it from the simplified model and performing LRTs. To obtain goodness of fit for our mixed models, we calculated the *r*^*2*^ of the relationship between the actual data and model-predicted values (De Vries *et al.*, 2012). The contribution of plant species identity to goodness of fit for our mixed models was estimated by subtracting the goodness-of-fit *r*^*2*^ for a model without species from the model with species. Statistical significance amongst the different species was obtained through formulating contrast statements within the same model structure, whilst controlling for multiple contrasts (see Hothorn *et al.*, 2008; Cichini *et al.*, 2011).

#### Microcosm root decomposition experiment analysis

We used principal component analysis (vegan package in R; Oksanen *et al.*, 2008) to simplify the analysis of root decomposition, and combined multiple measures of decomposition into a single term. A single root decomposition measure was achieved using the first axis scores, combining mass and C loss (g g^−1^); CO_2_-C efflux (μmol CO_2_-C g^−1^ root C min^−1^) and phenol peroxidase activity (μmol diqc g^−1^ min^−1^), which explained 92.2% of the variation across species (Supporting Information, Fig. S1). However, N loss correlated poorly with the other measures of decomposition and was analysed separately. Individual regression analyses were used to explore root traits as predictors of species differences in decomposition (first axis scores) and loss of N from roots. Individual regressions were used as opposed to a multiple regression, because of strong collinearity between root traits (Table S1). *R. acris* root decomposition exceeded that of all other microcosm species (125% above the other species mean loss of mass and C from roots); therefore decomposition measures in the absence of *R. acris* were analysed separately following the procedure outlined earlier (Fig.S1; Table S1).

## Results

### Livestock grazing effects on root decomposition

**Fig 1:**
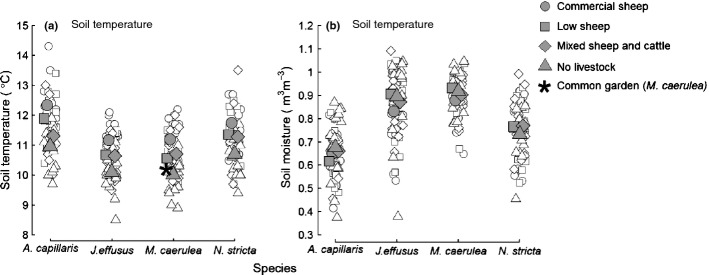
Soil temperature (a) and moisture (b) for spot measurements at the point where individual litterbags were buried under four species swards (*Agrostis capillaris*, *Juncus effusus*, *Molinia caerulea*, *Nardus stricta*) for the main grazing experiment. All litterbag points are shown as white symbols. Grazing treatments are indicated in the key. Mean soil temperature and moisture for each grazing treatment are shown in corresponding larger grey-filled symbols. The asterisk is the mean soil temperature and soil moisture in the common-garden experiment (*M. caerulea-*dominated) where litterbags of all four species were buried.

**Fig 2:**
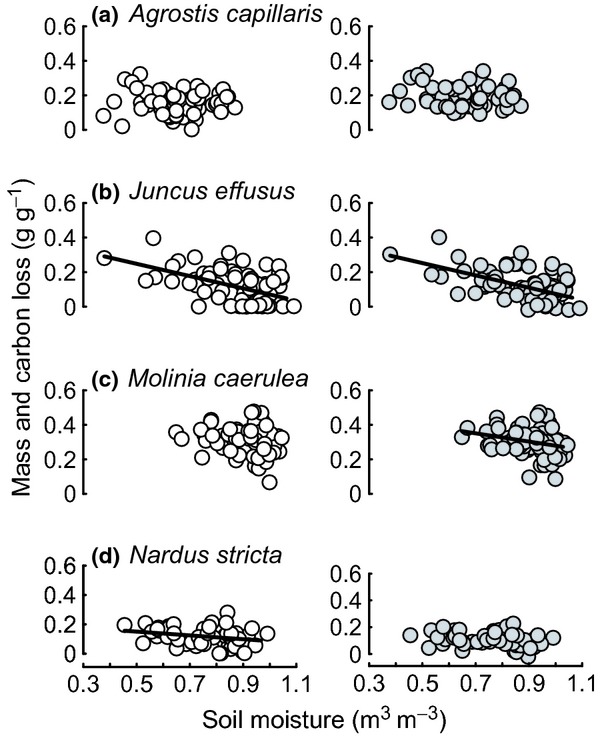
Plots of root decomposition against variation in soil moisture of plant species swards for all grazing treatments: (a) *Agrostis capillaris*; (b) *Juncus effusus*; (c) *Molinia caerulea*; (d) *Nardus stricta*. Root mass loss (g g^−1^), white circles; carbon loss (g g^−1^), grey circles. Significant linear mixed-effect model fits are shown for each species with a solid line.

Soils were significantly warmer under more intense livestock grazing in swards of all four dominant upland grass species (Fig. 1; Table 1), with the greatest treatment differences in *A. capillaris* swards (1.38 ± 0.16°C (± 1 SD) warmer under commercial than under no grazing). However, differences in soil temperature did not significantly impact any measure of root decomposition (Table 1). Instead, increasing soil moisture significantly reduced mass and C loss from root litter (Fig. 2), but livestock grazing did not affect soil moisture (Fig. 1; Table 1).

**Table 1:** Summary statistics for soil temperature, soil water, mass loss, carbon (C) loss, CO_2_-C efflux and nitrogen (N) loss from root litter

Factor	Soil temperature			Soil moisture			Mass loss (g g^−1^)			Carbon loss (g g^−1^)			CO_2_-C efflux (μmol CO_2_-C g^−1^ root C min^−1^)			Nitrogen loss (g g^−1^)		
	*χ*^2^	df	*P*	*χ*^2^	df	*P*	*χ*^2^	df	*P*	*χ*^2^	df	*P*	*χ*^2^	df	*P*	*χ*^2^	df	*P*
Grazing treatment	27.07	3	< 0.001	–	–	–	–	–	–	–	–	–	–	–	–	–	–	–
Plant species	77.18	16	< 0.001	83.28	3	< 0.001	283.1	6	< 0.001	253.91	6	< 0.001	–	–	–	174.57	3	< 0.001
Soil temperature	–	–	–	18.29	1	< 0.001			–	–	–	–	29.31	13	0.006	–	–	–
Soil moisture	27.22	14	0.018	–	–	–	63.67	4	< 0.001	28.0	4	< 0.001	–	–	–	–	–	–
Tographical exposure (Topex)	12.31	1	< 0.001	–	–	–	–	–	–	–	–	–	–	–	–	–	–	–
Plant species × soil moisture	–	–	–	–	–	–	7.48	3	0.058	10.66	3	0.014	–	–	–	–	–	–
Species variance explained (%)	7.40			11.19			64.17			66.89			-			51.94		
Total variance explained (%)	78.23			53.97			68.82			66.93			21.14			51.94		

Final models shown have been simplified using Akaike's information criterion (AIC) and retained if found to be significant following *χ*^2^ likelihood ratio deletion test. For each factor, *χ*^2^ values, associated degrees of freedom and *P*-values are shown when removed from the final selected model. Total variance explained is a measure of goodness of fit for mixed models, calculated from the *r*^2^ of the relationship between the actual data and model-predicted values (De Vries *et al.*, 2012). Species variance explained within each model was obtained by subtracting the *r*^2^ goodness of fit for the final model from a model without species.

Identity of the plant species (root litter used, effect of living sward, or a combination of both) explained the majority of variation (66.9–68.8%) in mass and C loss from roots, followed by soil moisture and the interaction between the two (Table 1; Fig. 2). However, only litter/sward identity significantly explained variation in root N loss (Table 1). The decrease in root C loss with increasing soil moisture under *J. effusus* was significantly greater than under *A. capillaris* (*z* = 2.82, *P* = 0.024) and marginally significantly greater than under *N. stricta* (*z* = 2.46, *P* = 0.065), but did not differ from *M. caerulea* (*z* = 0.90, *P* = 0.804). A similar (Fig. 2) plant species × soil moisture interaction driven by *J. effusus* was seen for root mass loss, but was only marginally significant overall (Table 1).

Plant species swards occupied different soil moisture niches; *A. capillaris* and *N. stricta* favoured drier outcrops, *M. caerulea* favoured wet mires, while *J. effusus* swards occurred across the full soil moisture gradient (Fig. 2). At the wet end of the moisture gradient, no root mass loss occurred in 16 *J. effusus* litterbags (out of 19 litterbags that did not lose mass for the entire experiment), while *M. caerulea* roots consistently lost mass and C.

### Relative effects of soil microclimate and root species identity on root decomposition

In the main field experiment, root litter was buried underneath its conspecific sward, while the common-garden experiment investigated the effect of root litter vs soil microclimate and plant sward effects. Average soil temperature in the *M. caerulea*-dominated common-garden experiment was similar (10.2°C) to ungrazed *M. caerulea* communities across the landscape (10.0°C), while soils were, on average, slightly drier (0.82 vs 0.92 m^3^ m^−3^; Fig. 1).

**Fig 3:**
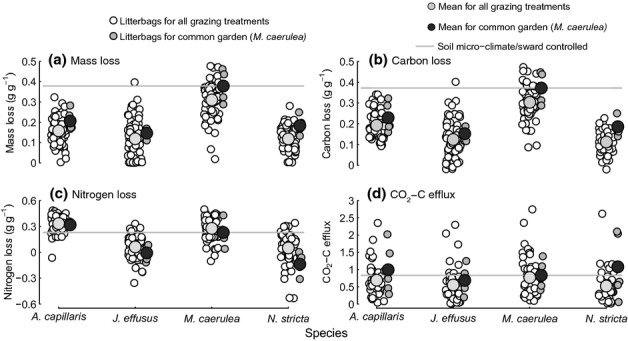
Loss of mass (a), carbon (b), nitrogen (c) and CO_2_-C efflux (d) from decomposing roots of four grass species: *Agrostis capillaris*, *Juncus effusus*, *Molinia caerulea* and *Nardus stricta*. Root decomposition for all litterbags is shown across the Glen Finglas grazing experiment (white circles) and the *M. caerulea-*dominated common-garden (grey circles) experiment, and species means for each experiment correspond to larger symbols (light grey circles for the main grazing experiment and dark grey circles for the common-garden experiment). The expected rates of root decomposition if soil microclimate (temperature and moisture) and/or the live species sward were the key controlling factors of root decomposition are represented by the solid grey lines, which are at the same rates of decomposition as the mean of common-garden *M. caerulea* roots.

Despite decomposing under similar soil microclimatic conditions, mass (*F*_3,16_ = 20.52, *P *< 0.001), C (*F*_3,16_ = 14.75, *P *< 0.001) and N loss from roots (*F*_3,16_ = 38.43, *P *< 0.001) differed significantly among species in the common-garden experiment (Fig. 3). Species root decomposition followed a similar pattern as in the main grazing experiment. In the common-garden experiment, *M. caerulea* roots lost the greatest amount of mass (0.376 g g^−1^ ± 0.142), having lost 45, 61 and 51% more than *A. capillaris*, *J. effusus* and *N. stricta*, respectively, and similar differences were seen across all treatments in the grazing experiment (Fig. 3a). C loss followed a similar pattern to mass loss in the common-garden and main grazing experiments (Fig. 3b). The pattern of N loss among species did not match root mass and C loss, but followed the same species pattern in both experiments (Fig. 3c).

There was no demonstrable effect of the live *M. caerulea* sward on root decomposition. Decomposition of each species was similar under *M. caerulea* to that under its conspecific sward, despite differences in soil environment associated with different dominant sward species (Fig. 3). CO_2_-C efflux was the only decomposition measure that did not differ between species in the common-garden (*F*_3,16_ = 0.350, *P* = 0.789) or the main experiment (Table 1). Instead CO_2_-C efflux from *ex situ* root litter was positively correlated with increasing field soil temperature (Table 1). Overall, root decomposition (mass, C and N loss) was determined by litter identity, rather than grazing-induced changes in soil microclimate or effects of the live plant sward.

### Root traits predicting decomposition

**Fig 4:**
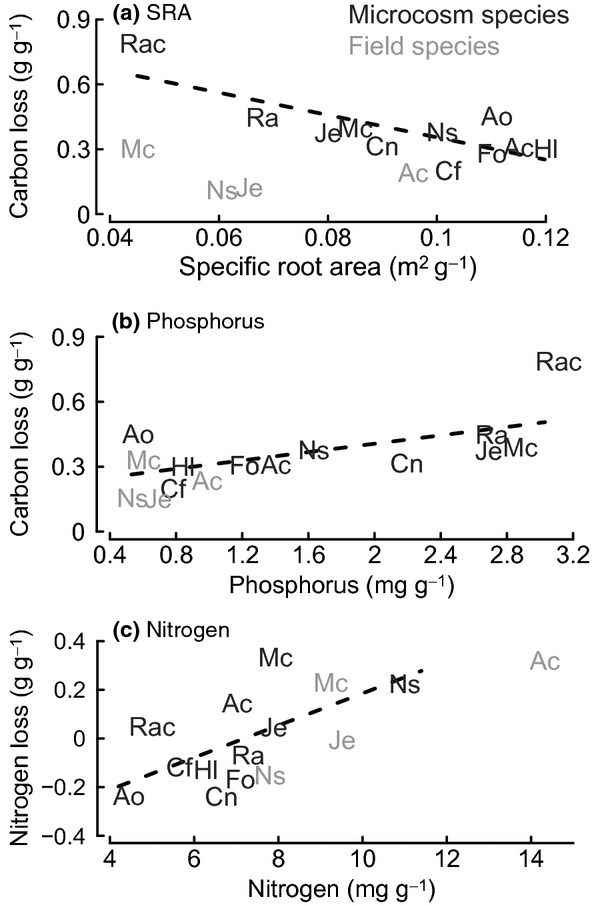
Root traits predicting root decomposition for microcosm species: (a) specific root area (SRA), that is, the surface area of the root per unit of mass (m^2^ g^−1^); (b) phosphorus content as predictors of loss of carbon from roots; and (c) nitrogen content as a predictor of loss of N from roots. Microcosm species roots are in dark grey with a dashed line for linear model fit for microcosm species only. Average traits and rates of root decomposition for field species from the main grazing experiment are in light grey. Species abbreviations: Ac, *Agrostis capillaris*; Ao, *Anthoxanthum odoratum*; Cf, *Cerastium fontanum*; Cn, *Carex nigra*; Fo, *Festuca ovina*; Hl, *Holcus lanatus*; Je, *Juncus effusus*; Mc, *Molinia caerulea*; Ns, *Nardus stricta*; Rac, *Ranunculus acris*; Ra, *Rumex acetosa*.

Specific root area was the strongest predictor of root decomposition in the laboratory microcosm experiment from the selection of root traits measured (Tables2, S2). Root decomposition (defined here as the principal component of root mass and C loss, *ex situ* CO_2_-C efflux and phenol peroxidase activity) was greater for species with a low SRA (Fig. 4). In microcosms, the SRA of *R. acris* roots was far smaller than that of any other species, yet SRA remained a significant predictor without *R. acris* in the statistical analysis (Table 2). In the field experiment, *M. caerulea* had the greatest loss of mass and C from roots and an SRA 61% lower than the mean of all the other species (Table 3).

**Table 2:** Chemical and morphological traits of undecomposed roots (means for all species ± 1 SD) as predictors of root decomposition (axis 1 scores for mass loss (g g^−1^), carbon (C) loss (g g^−1^), CO_2_-C efflux (μmol CO_2_-C g^−1^ root C min^−1^) and phenol peroxidase activity (μmol diqc g^−1^ min^−1^)) and nitrogen loss (g g^−1^) for all 11 upland grassland species, including *Ranunculus acris* (+Rac) (grey text)

Traits	Mean trait		Decomposition (PCA axis 1 [*r*^2^])		Nitrogen loss (*r*^2^)	
	+Rac	−Rac	+Rac	−Rac	+Rac	−Rac
Chemical traits						
N (mg g^–1^)	6.91 (1.76)	7.10 (1.73)	0.10	0.04	**0.37**[Table-fn tf2-1]*	**0.49**[Table-fn tf2-1]*
Ca (mg g^–1^)	0.79 (0.42)	0.78 (0.44)	0.01	0.20	0.06	0.07
K (mg g^–1^)	6.96 (3.44)	6.48 (3.23)	0.25	0.07	0.11	0.10
P (mg g^–1^)	1.81 (3.44)	1.69 (0.86)	**0.34**[Table-fn tf2-1]**•**	**0.33**[Table-fn tf2-1]**•**	0.19	0.19
Si (mg g^–1^)	9.99 (4.49)	10.80 (3.78)	**0.39**[Table-fn tf2-1]*	0.04	0.02	0.01
C : N ratio	70.4 (15.7)	65.6 (15.2)	0.14	0.01	**0.35**[Table-fn tf2-1]**•**	**0.49**[Table-fn tf2-1]*
Lignin : N ratio	27.8 (7.9)	27.8 (7.9)	0.01	0.01	0.01	0.01
Morphological traits						
Root diameter (mm)	0.27 (0.04)	0.26 (0.04)	0.13	0.02	0.16	0.22
SRA (m^2^ g^−1^)	0.09 (0.02)	0.10 (0.02)	0.64**	0.41*	0.04	0.03
SRL (m g^−1^)	11.3 (4.1)	12.0 (3.7)	**0.31**•	0.03	0.01	0.03
RDMC (g g^−1^)	0.19 (0.21)	0.19 (0.22)	0.01	0.01	**0.34**•	**0.34**•
Litterbag bulge (cm g^−1^)	2.59 (0.53)	2.69 (0.43)	0.46*	0.05	0.01	0.01
Mycorrhiza colonization (%)	9.16 (9.81)	9.19 (10.35)	0.01	0.07	0.01	0.01

The same predictions are made for 10 species without *R. acris* (−Rac) (black text). Significant root traits are shown in bold and denoted as follows:

• , *P *< 0.1

* , *P *< 0.05

** , *P *< 0.01.

All other *r*^*2*^-values were not significant.

PCA, principal component analysis; SRA, specific root area; SRL, specific root length; RDMC, root dry matter content.

**Table 3:** Chemical and morphological traits of undecomposed roots of four dominant upland grassland species collected from the main grazing experiment

Traits	Species			
	*Agrostis capillaris*	*Juncus effusus*	*Molinia caerulea*	*Nardus stricta*
Chemical traits				
C (mg g^–1^)	437.9 (19.8)	451.1 (22.8)	462.5 (5.4)	435.9 (40.8)
N (mg g^–1^)	14.3 (2.1)	9.5 (2.6)	9.24 (9.0)	7.39 (6.47)
Ca (mg g^–1^)	3.79 (0.12)	3.08 (0.08)	2.24 (0.10)	4.85 (0.12)
P (mg g^–1^)	0.90 (0.01)	0.61 (0.02)	0.50 (0.07)	0.53 (0.11)
Si (mg g^–1^)	15.2 (2.2)	12.3 (0.3)	9.3 (0.2)	15.1 (0.2)
C : N ratio	32.8 (5.1)	49.4 (15.1)	52.4 (13.0)	67.5 (7.1)
Lignin : N	2.65 (0.60)	5.12 (0.61)	3.74 (0.40)	5.32 (0.58)
Morphological traits				
Root diameter (mm)	0.22 (0.08)	0.32 (0.03)	0.35 (0.07)	0.26 (0.01)
Specific root area (m^2^ g^−1^)	0.10 (0.01)	0.07 (0.01)	0.05 (0.01)	0.06 (0.01)
Specific root length (m g^−1^)	139.2 (13.5)	70.3 (16.8)	44.2 (21.2)	74.0 (10.5)
Root dry matter content (g g^−1^)	0.18 (0.02)	0.16 (0.02)	0.18 (0.02)	0.18 (0.03)
Mycorrhiza colonization (%)	44.8 (4.5)	0	24.6 (8.3)	21.4 (6.5)

All traits are means per species (± 1 SD).

Initial root P was a marginally significant predictor of decomposition, both with and without *R. acris* (Table 2). Higher initial root P predicted greater decomposition, with *R. acris* roots containing 184% more P than the mean of the other species. Positive effects of initial root P were not apparent in roots decomposing in the main field experiment, as concentrations and their range among the four species were small compared with those observed in the pot-grown roots (Table 3; Fig. 4). Root silica content, SRL and the index of the initial volume of litterbags (‘bulge’) were not consistent root decomposition predictors, as their significance was driven by *R. acris* (Table 2). The root trait measures P and SRA were significantly and negatively correlated with one another, and root silica was positively correlated with SRA, SRL and litterbag ‘bulge’ (Table S1). None of the other root traits measured were significant individual predictors of root decomposition, despite substantial variation in root quality among species (e.g. N, C and K; Table 2). Phenol peroxidase enzyme activity, in the absence of *R. acris*, was only predicted by root Ca content; this positive correlation was driven by a single species, *C. fontanum*, which contained 275% more Ca than the other species.

Significant predictors of N loss from roots differed from the other measures of decomposition, with higher initial root N content predicting greater N loss (Table 2). Loss of root N was significantly and negatively correlated with root C : N ratio and marginally significantly negatively correlated with root dry matter content (Table 2). In microcosms, roots of some species lost N, while most – with an initial N content below 7 mg g^−1^ – gained N during decomposition (Fig. 4). Roots decomposing in the main field experiment followed this pattern, with the very high initial N content of *A. capillaris* roots losing the most N and low initial N of *J. effusus* and *N. stricta* gaining N (Fig. 4; Table 3). Root N traits (N, C : N, lignin : N) failed to predict any other measure of root decomposition besides loss of root N from microcosm species (Table 2).

## Discussion

**Fig 5:**
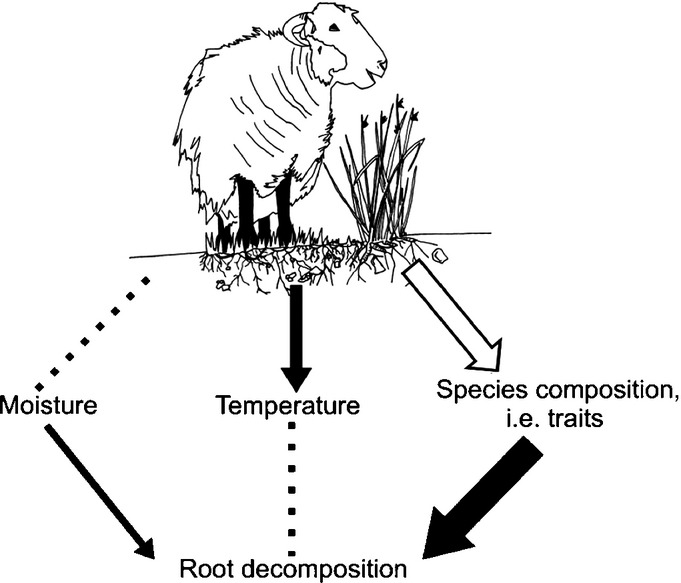
Conceptual diagram of the impact of livestock grazing on root decomposition, through grazing effects on soil microclimate (moisture and temperature) and species composition (i.e. root traits). Closed black arrows, significant direct effects (larger arrows indicate the increasing strength of that effect); dotted lines, measured nonsignificant direct effects; open arrow, unmeasured direct effects.

Although several studies have identified the importance of plant traits in explaining variation in microbial community composition at the landscape scale (De Vries *et al.*, 2012) and leaf litter decomposition across varying intensities of land management (Garnier *et al.*, 2004; Fortunel *et al.*, 2009), our study provides new insights into how variation in plant traits acts on decomposition of root litter at the landscape scale and how this is mediated by grazing management practices. We used three complementary approaches to disentangle the potential effects of large herbivores on below-ground decomposition via possible changes in soil temperature, moisture and species composition (traits), thereby identifying the importance of litter identity in driving root decomposition. By quantifying root decomposition from 11 plant species in a controlled environment microcosm experiment, we identified specific traits that can predict root decomposition. In our upland grassland system, rates of root decomposition were dependent on litter identity and the underlying root traits – SRA and P concentration. Our results suggest that below-ground C and N dynamics in these upland grasslands will depend more on changes in plant species composition than on grazing-induced changes in soil microclimate (Fig. 5).

Lack of sensitivity of decomposing roots to a range of soil microclimate conditions has been attributed to the soil buffering roots and microbes from abiotic extremes (Silver & Miya, 2001; McLaren & Turkington, 2010). Our results suggest that the soil environment, particularly soil moisture, inhibits decomposition, reducing loss of mass and C from roots for all four focal plant species across the landscape. In peaty and podzolic soils, negative effects of soil moisture on decomposition are often a result of low oxygen availability and pH, limiting microbial abundance, extracellular enzyme activity and diversity (Papanikolaou *et al.*, 2010). Roots from warmer soils had greater *ex situ* CO_2_-C efflux, suggesting that the indirect effect of grazing-induced warming may have influenced the microbial community, but that microbial activity was constrained by the high soil moisture content in the field. Despite respiring more CO_2_-C, roots from warmer soil lost mass and C at similar rates to roots from cooler soils. An alternative explanation for greater microbial activity without loss of mass or C from roots may be that, in more intensely sheep-grazed swards with warmer soil, microbes had access to alternative C sources such as animal excreta, sloughed roots or root and fungal exudates (Bardgett *et al.*, 1998; Wardle *et al.*, 2004; Klumpp *et al.*, 2009). Thus, although grazing potentially affects the microbial community, it did not significantly affect loss of mass, C and N from decomposing roots, either directly or through changes in soil temperature.

Loss of mass and C from roots could be partially attributed to differences in soil moisture across the landscape under the different plant communities. *J. effusus* had the greatest range in root mass and C loss and the greatest range in soil moisture occupied by the species sward, occurring on drier soils with *A. capillaris* and in wetter *M. caerulea*-dominated mires (Rodwell, 1991, 1992). However, in wetter mires (> 0.92 m^3^ m^−3^) *J. effusus* roots did not lose mass or C, while in similar conditions *M. caerulea* roots continued to decompose. This difference may be a result of the effect of the live plant community on soil micro-organisms as seen in other grasslands (Johnson *et al.*, 2004; Weiss *et al.*, 2005; Neubauer *et al.*, 2007). However, given the overriding significance of root litter type in all our experiments (Fig. 5), differences in root decomposition between the two species across a soil moisture gradient are likely to be a result of root litter identity. In the *M. caerulea*-dominated common-garden experiment, under shared soil moisture conditions, loss of mass and C from *M. caerulea* roots remained significantly greater than from *J. effusus* roots. Litter identity, as opposed to the live plant community, regulates leaf litter decomposition (Trinder *et al.*, 2009; Coq *et al.*, 2011) and this also seems to be true for decomposing roots in our upland grassland system.

Species differences in root decomposition were significantly correlated with root traits. The principal explanatory trait was SRA, the root surface area per unit of mass. Smaller SRA correlated with greater root decomposition, and species with low SRA were *M. caerulea* (in the field) and *R. acris* (in microcosms). This is counterintuitive as roots with a smaller external surface area should have less area accessible for micro-organisms to colonize and decompose. Decomposition of tree roots has similarly been found to correlate negatively with SRL, as thicker roots decompose quicker than thinner roots in the initial 6 months, but this reverses into a positive correlation after 18 months (Hobbie *et al.*, 2010). Therefore, the negative relationship between decomposition and SRA may only be a short-term phenomenon. However, the majority of our microcosm species' root litter mass loss (25–77%) occurred in the initial 6 months. The negative correlation between SRA and decomposition is therefore important and may be explained, to some extent, by a greater internal surface in thicker roots as a result of aerenchyma (air spaces) (Thormann *et al.*, 2000). However, aerenchyma cannot completely explain the relationship between SRA and root decomposition rates, because some faster (e.g. *M. caerulea* and *R. acris)* and slower (e.g. *J. effusus* and *N. stricta*) species form aerenchyma when flooded (Smirnoff & Crawford, 1983; Justin & Armstrong, 1987; Lloyd *et al.*, 1998), and in our field experiment, decomposition was slower in wetter environments. Alternatively, thicker roots may contain more large cortical storage cells in the root periphery, as seen in *M. caerulea* (Jefferies, 1916), which would be easily accessed by decomposing microbes (Robinson, 1990). As the number of traits used to predict root decomposition remains limited (Cornelissen *et al.*, 2003; De Deyn *et al.*, 2008; Orwin *et al.*, 2010), further investigation into the relationship between SRA and root decomposition is required.

Previously identified predictors of root decomposition, including N content, lignin : N or C : N ratio, Ca concentrations and mycorrhizal colonization (Silver & Miya, 2001; Langley *et al.*, 2006; Hobbie *et al.*, 2010; Birouste *et al.*, 2012), did not correlate with loss of mass or C from roots in our study. Other studies have also found that N-related root traits did not predict species differences in root mass loss (Hobbie *et al.*, 2010; Freschet *et al.*, 2012a). Initial root N only predicted loss of N from roots, and this was predicted by the initial C : N ratios and root dry matter content to a lesser extent. The positive relationship between initial N concentration and N loss included roots in the microcosm and field experiments, with some species with low initial N content (< 7 mg g^−1^) gaining N during the incubation period. This follows the C-use efficiency hypothesis: litter degradation microorganisms with a higher N demand will uptake N from the soil (immobilizing N in litter) when decomposing N-impoverished substrates (Manzoni *et al.*, 2008). Initial root N concentrations are a function of edaphic properties and plant-available N during root growth (Robinson & Rorison, 1988). The significance of initial substrate quality determining root traits was particularly evident for *A. capillaris*; roots collected from the field had a higher N content and N loss than pot-grown roots. This is probably a result of the species preferring nutrient-rich soil and gaining N input (urine and faeces) from sheep. Plants under different grazing intensities would be expected to differ in root litter quality as a result of nutrient allocation and animal nutrient inputs (see Bardgett *et al.*, 1998) and this provides another pathway whereby grazing could influence plant traits and root decomposition (Fig. 5). As decomposition is dependent on initial root quality, identification of predictive traits requires plants to be grown in uniform conditions (Cornelissen *et al.*, 2003). Yet, to understand the response of decomposition to land-use management requires the use of roots shaped by the range of soil physicochemical conditions and management intensities in the field.

As found in our experiments, root P has been shown to be a significant predictor of root decomposition in microcosms (Birouste *et al.*, 2012), but not in field conditions (Moretto *et al.*, 2001; Hobbie *et al.*, 2010). Initial root P concentrations in the field, ranging from 0.5 to 0.9 mg g^−1^, were similar to other temperate grassland roots, ranging from 0.4 to 0.6 mg g^−1^ (Heal & Perkins, 1978; Van Vuuren *et al.*, 1993). On the other hand, P concentrations for pot-grown roots were potentially artificially high (ranging from 0.6 to 3.1 mg g^−1^; Table S2). This could have been a result of greater P availability when rearing plants; alternatively, there is evidence that P can be recycled from dying root cortical cells (Robinson, 1990), and the proportion of dead or dying roots in 6-month-old pot-grown plants would have been smaller than in the field. Live roots are commonly used to study root decomposition under the presumption that senescing roots do not undergo nutrient resorption (Aerts *et al.*, 1992; Freschet *et al.*, 2013), although caution should be taken when drawing conclusions using certain live root chemical traits to explain root decomposition. Nevertheless, given sufficient variation, initial root P can predict root decomposition, in a manner that is analogous to leaf P predicting leaf decomposition (Cornwell *et al.*, 2008; Orwin *et al.*, 2010; Birouste *et al.*, 2012). Our results suggest that, if roots are integrated into litter decomposition models (Manzoni *et al.*, 2010; Freschet *et al.*, 2013), SRA and initial root P, rather than N, will be better predictors of mass and C loss from root litter.

In summary, root decomposition depends on litter type and quality rather on than grazing-induced changes in the soil environment, including temperature. Loss of mass and C from roots varies with soil moisture across the landscape and thus the preferred hydrological niche occupied by the plant species sward. The lack of a moisture-mediated effect of grazing on root decomposition contradicts those models proposing that grazing in wetter ecosystems increases root C storage through effects on the decomposition pathway (Piñeiro *et al.*, 2010). Our results indicate an alternative interpretation, namely that changes in species composition and associated traits have greater influence on root decomposition than soil moisture (Fig. 5). Grazing management alters the species composition of upland grasslands over annual to decadal timescales (Ross *et al.*, 2012; Smith *et al.*, 2014). Long-term increases in *M. caerulea* and *N. stricta*, at the expense of *A. capillaris*, have been recorded in upland grasslands (Ross *et al.*, 2012) and, given their root trait differences, this will have significant implications for below-ground C and N dynamics.

## Supporting Information

Fig S1 Biplot of root decomposition measures mass and carbon loss CO_2_-C efflux; phenol peroxidase activity excluding loss of N from roots for 11 upland species and root decomposition measures excluding loss of N from roots and phenol peroxidase activity for 10 species without *Ranunculus acris*. **Table S1** Spearman's rank correlation coefficients of paired chemical and morphological traits for undecomposed roots of 11 upland grassland species and 10 species without *Ranunculus acris* **Table S2** Chemical and morphological traits of undecomposed roots for 11 upland grassland species commonly found in *Agrostis capillaris*–dominated upland grassland communities (germinated from seed, pot-grown for 6 months)
