# Supplementary material for: Root traits predict decomposition across a landscape-scale grazing experiment
Source: New Phytol. 2014 May 20;203(3):851–62. doi: 10.1111/nph.12845 (PMC4260134; doi:10.1111/nph.12845)
Supplement: Fig S1 — Biplot of root decomposition measures mass and carbon loss CO2-C efflux; phenol peroxidase activity excluding loss of N from roots for 11 upland species and root decomposition measures excluding loss of N from roots and phenol peroxidase activity for 10 species without Ranunculus acris. Table S1 Spearman's rank correlation coefficients of paired chemical and morphological traits for undecomposed roots of 11 upland grassland species and 10 species without Ranunculus acris Table S2 Chemical and morphological traits of undecomposed roots for 11 upland grassland species commonly found in Agrostis capillaris–dominated upland grassland communities (germinated from seed, pot-grown for 6 months) [file nph0203-0851-SD1.docx]

## Supporting Information Fig. S1 & Tables S1 and S2


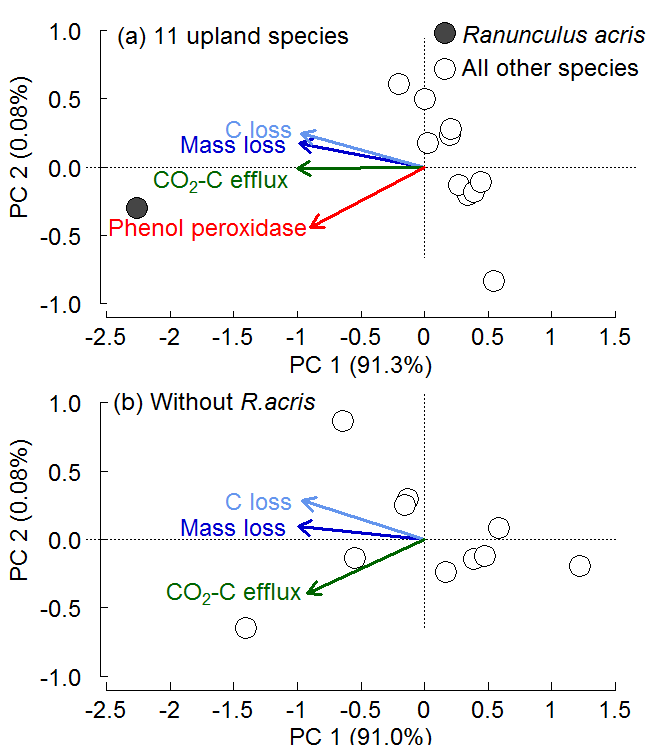


**Fig. S1** Biplot of (a) root decomposition measures mass and carbon loss (g g^-1^) CO_2_-C efflux (µmol gC^-1^min^-1^); phenol peroxidase activity (µmol diqc. g^-1^ min^-1^) excluding loss of N from roots for 11 upland species and (b) root decomposition measures excluding loss of N from roots and phenol peroxidase activity for 10 species without *Ranunculus acris*. First axis scores from both redundancy analyses were used as a single measure of decomposition in the root trait analysis. *Ranunculus acris* is the dark grey symbol, while all other species in the analysis are white symbols.

**Table S1** Spearman’s rank correlations coefficients of paired chemical and morphological traits for un-decomposed roots .Values range from -1, through 0, to 1 describe a perfect negative, through no correlation, to perfect positive correlation. Correlation coefficients for 11 upland grassland species commonly found in *Agrostis capillaris*–dominated upland plant communities (germinated from seed and pot-grown for 6 months) are in grey text, whereas correlation coefficients for 10 species, excluding *Ranunculus acris,* are in black text. Significant colineratiy between root traits is shown in bold and denoted: •, *P*<0.1; *, *P*<0.05; **, *P*<0.01; ***, *P*<0.001, all other correlation coefficient scores were not significant.

| Root traits |  | N  (mg g^-1^) | Ca  (mg g^-1^) | K  (mg g^-1^) | P  (mg g^-1^) | Si  (mg g^-1^) | C:N  ratio | Lignin:N  ratio | Root diameter (mm) | Specific root area  (m^2^ g^-1^) | Specific root length  (m^2^ g^-1^) | Root dry matter content  (g g^-1^) | Litterbag bulge  (cm g^-1^) | Myccorhiza colonization  (%) | |
| --- | --- | --- | --- | --- | --- | --- | --- | --- | --- | --- | --- | --- | --- | --- | --- |
| N |  |  | 0.01 | 0.27 | 0.45 | -0.09 | **-0.98***** | -0.03 | -0.32 | -0.25 | 0.15 | -0.38 | 0.21 | -0.06 | With *Ranunuculus acris* |
| Ca | Without *Ranunculus acris* | -0.07 |  | 0.43 | 0.13 | **-0.72*** | -0.04 | -0.18 | **0.53•** | -0.43 | -0.62 | 0.34 | -0.18 | **-0.59•** |  |
| K |  | **0.58•** | 0.38 |  | **0.81**** | **-0.71*** | -0.31 | **-0.57•** | 0.46 | **-0.83**** | **-0.65*** | -0.07 | -0.30 | **-0.54•** |  |
| P |  | 0.81 | 0.01 | **0.75*** |  | **-**0.46 | -0.45 | -0.15 | 0.24 | **-0.83**** | -0.51 | -0.26 | -0.36 | -0.14 |  |
| Si |  | -0.33 | **-0.73*** | **-0.61•** | -0.28 |  | 0.13 | 0.44 | -0.40 | **0.70*** | **0.74**** | -0.31 | **0.60•** | 0.50 |  |
| C:N |  | **-0.98***** | -0.09 | **-0.62•** | **-0.81**** | 0.38 |  | 0.15 | 0.32 | 0.27 | -0.10 | 0.31 | -0.15 | 0.16 |  |
| Lignin: N |  | -0.08 | -0.18 | **-0.64*** | -0.15 | 0.45 | 0.22 |  | -0.16 | 0.22 | 0.22 | -0.19 | 0.21 | **0.62*** |  |
| Root diameter |  | -0.36 | 0.48 | 0.36 | 0.10 | -0.33 | 0.36 | -0.16 |  | -0.49 | **-0.68*** | 0.47 | -0.24 | **-0.60•** |  |
| SRA |  | **-0.57•** | -0.35 | **-0.77**** | **-0.78**** | **0.60•** | **0.60•** | 0.26 | -0.40 |  | **0.87***** | -0.15 | 0.39 | **0.53•** |  |
| SRL |  | -0.07 | **-0.56•** | -0.54 | -0.35 | **0.65*** | 0.13 | 0.24 | **-0.67*** | **0.82**** |  | -0.43 | 0.50 | **0.65*** |  |
| RDMC |  | -0.43 | 0.37 | 0.01 | -0.21 | -0.44 | 0.35 | -0.21 | 0.54 | -0.29 | -0.61 |  | -0.31 | **-0.54•** |  |
| Litterbag bulge |  | 0.05 | -0.07 | -0.07 | -0.15 | 0.47 | 0.03 | 0.18 | -0.18 | 0.19 | 0.33 | -0.42 |  | 0.04 |  |
| Mycorrhiza colonization |  | -0.04 | **-0.61•** | **-0.70*** | -0.26 | **0.65*** | 0.16 | **0.68*** | **-0.61•** | **0.68*** | **0.82**** | **-0.58•** | 0.14 |  |  |

**Table S2** Chemical and morphological traits of un-decomposed roots for 11 upland grassland species commonly found in *Agrostis capillaris*–dominated upland grassland communities (germinated from seed, pot-grown for 6 months). All traits are means per species (± 1 SD).

|  | Trait | Species | | | | | | | | | | |
| --- | --- | --- | --- | --- | --- | --- | --- | --- | --- | --- | --- | --- |
|  |  | *Agrostis capillaris* | *Anthoxanthum odoratum* | *Cerastium fontanum* | *Carex nigra* | *Festuca ovina* | *Holcus lanatus* | *Juncus effusus* | *Molinia caerulea* | *Nardus stricta* | *Ranunculus acris* | *Rumex acetosa* |
| Chemical traits | C (mg g^-1^) | 476.88  (5.43) | 454.43  (2.78) | 414.65  (4.42) | 477.17  (7.02) | 481.36  (11.93) | 467.95  (7.10) | 464.35  (11.91) | 464.68  (7.84) | 478.68  (10.06) | 469.67  (5.63) | 445.23  (4.17) |
|  | N (mg g^-1^) | 7.02  (0.18) | 4.45  (0.18) | 5.65  (0.16) | 6.65  (0.24) | 7.08  (0.17) | 6.08  (0.17) | 7.89  (0.28) | 7.93  (0.13) | 11  (0.21) | 7.28  (0.28) | 5  (0.11) |
|  | Ca (mg g^-1^) | 0.5  (0.10) | 0.42  (0.02) | 1.87  (0.12) | 0.7  (0.04) | 0.82  (0.16) | 0.51  (0.02) | 0.94  (0.08) | 0.33  (0.02) | 0.76  (0.03) | 0.94  (0.03) | 0.9  (0.12) |
|  | K (mg g^-1^) | 2.22  (0.19) | 3.98  (2.98) | 6.25  (0.05) | 10.3  (0.52) | 3.74  (0.04) | 2.39  (0.42) | 10.18  (0.30) | 7.49  (0.34) | 10.05  (0.22) | 8.24  (0.35) | 11.7  (0.63) |
|  | P (mg g^-1^) | 1.4  (0.07) | 0.57  (0.02) | 0.78  (0.01) | 2.2  (0.11) | 1.21  (0.27) | 0.84  (0.01) | 2.75  (0.18) | 2.76  (0.07) | 1.63  (0.12) | 2.71  (0.13) | 3.11  (0.13) |
|  | Si (mg g^-1^) | 12.14  (2.80) | 17.13  (4.85) | 7.74  (5.34) | 10.11  (3.33) | 10.17  (1.84) | 15.34  (6.60) | 11.07  (5.63) | 11.37  (3.96) | 9.63  (2.81) | 3.46  (1.06) | 1.83  (0.84) |
|  | C: N ratio | 102.25  (4.71) | 67.94  (1.83) | 73.45  (1.66) | 71.82  (1.60) | 67.98  (0.11) | 76.95  (1.46) | 58.87  (1.31) | 58.6  (0.10) | 43.5  (0.11) | 64.56  (2.15) | 89.04  (1.86) |
|  | Lignin (% mass) | 13.74  (3.72) | 15.83  (4.29) | 14.65  (2.13) | 10.2  (2.73) | 27.26  (4.40) | 23.27  (1.59) | 22.16  (1.76) | 28.31  (1.08) | 17.9  (2.95) | 20.32  (2.70) | 13  (3.89) |
| Morphological traits | Root diameter (mm) | 0.23  (0.02) | 0.24  (0.10) | 0.28  (0.04) | 0.32  (0.03) | 0.23  (0.01) | 0.25  (0.02) | 0.31  (0.02) | 0.23  (0.01) | 0.22  (0.01) | 0.31  (0.01) | 0.31  (0.02) |
|  | SRA (m^2^ g^-1^) | 0.115  (0.005) | 0.111  (0.007) | 0.102  (0.092) | 0.09  (0.005) | 0.111  (0.005) | 0.12  (0.005) | 0.081  (0.006) | 0.084  (0.012) | 0.101  (0.010) | 0.068  (0.013) | 0.046  (0.006) |
|  | SRL (m^2^ g^-1^) | 16.16  (1.61) | 14.51  (1.29) | 7.2  (13.06) | 9.14  (0.50) | 15.37  (0.70) | 15.52  (1.32) | 8.39  (0.95) | 11.79  (1.79) | 14.87  (1.09) | 6.93  (1.35) | 4.86  (0.80) |
|  | RDMC (g g^-1^) | 0.2  (0.03) | 0.21  (0.03) | 0.21  (0.05) | 0.2  (0.03) | 0.2  (0.02) | 0.15  (0.02) | 0.18  (0.02) | 0.17  (0.02) | 0.16  (0.01) | 0.22  (0.03) | 0.18  (0.01) |
|  | Litterbag bulge (cm g^-1^) | 3.03  (0.38) | 2.63  (0.21) | 2.16  (0.26) | 2.82  (0.24) | 2.94  (0.30) | 2.92  (0.35) | 3.13  (0.35) | 2.6  (0.45) | 2.95  (0.30) | 1.76  (0.43) | 1.55  (0.24) |
|  | Mycorrhiza colonization (%) | 11.58  (5.09) | 8.63  (7.71) | 0 | 0 | 17.2  (8.55) | 32.37  (6.31) | 0 | 13.58  (6.33) | 8.59  (11.30) | 0 | 8.86  (1.61) |
